# A comparison of interventional clinical trials in rare versus non-rare diseases: an analysis of ClinicalTrials.gov

**DOI:** 10.1186/s13023-014-0170-0

**Published:** 2014-11-26

**Authors:** Stuart A Bell, Catrin Tudur Smith

**Affiliations:** Department of Biostatistics, University of Liverpool, Duncan Building, Daulby Street, Liverpool, L69 3GA UK

**Keywords:** Rare disease clinical trial, ClinicalTrials.gov

## Abstract

**Objectives:**

To provide a comprehensive characterisation of rare disease clinical trials registered in ClinicalTrials.gov, and compare against characteristics of trials in non-rare diseases.

**Design:**

Registry based study of ClinicalTrials.gov registration entries.

**Methods:**

The ClinicalTrials.gov registry comprised 133,128 studies registered to September 27, 2012. By annotating medical subject heading descriptors to condition terms we could identify rare and non-rare disease trials. A total of 24,088 Interventional trials registered after January 1, 2006, conducted in the United States, Canada and/or the European Union were categorised as rare or non-rare. Characteristics of the respective trials were extracted and summarised with comparative statistics calculated where appropriate.

**Main outcome measures:**

Characteristics of interventional trials reported in the database categorised by rare and non-rare conditions to allow comparison.

**Results:**

Of the 24,088 trials categorised 2,759 (11.5%) were classified as rare disease trials and 21,329 (88.5%) related to non-rare conditions. Despite the limitations of the database we found that rare disease trials differed to non-rare disease trials across all characteristics that we examined. Rare disease trials enrolled fewer participants (median 29 vs. 62), were more likely to be single arm (63.0% vs. 29.6%), non-randomised (64.5% vs. 36.1%) and open label (78.7% vs. 52.2%). A higher proportion of rare disease trials were terminated early (13.7% vs. 6.3%) and proportionally fewer rare disease studies were actively pursuing, or waiting to commence, enrolment (15.9% vs. 38.5%).

**Conclusion:**

Rare disease interventional trials differ from those in non-rare conditions with notable differences in enrolment, design, blinding and randomisation. However, clinical trials should aim to implement the highest trial design standards possible, regardless of whether diseases are rare or not.

## Background

In the United States (US), a rare disease is defined as having a prevalence of fewer than 200,000 affected individuals [1]. Across the European Union (EU) the definition is that the condition affects not more than 5 in 10,000 individuals [2]. The Orpha.net database, which provides a reference portal for information on rare diseases, identifies approximately 7,000 rare diseases [3].

It is often assumed that clinical trials in rare conditions differ from those of non-rare conditions. However, the extent and nature of these differences is not well understood. Kesselheim *et al.* provide one comparative survey exploring pivotal trials in orphan versus non-orphan drug approval [4]. These authors characterised a number of preapproval trials highlighting differences in enrolment, randomisation, blinding, comparison groups and primary outcomes. However, their survey was limited to oncology trials that supported successful drug approvals. The approach of Kesselheim *et al.* was extended by Orfali *et al.* to clinical trials of non-oncological orphan drugs compared with those of non-orphan drugs [5]. These authors concluded that characteristics such as blinding, randomisation and placebo control were similar between trials of orphan vs. non-orphan drugs. However, methodological flaws in their approach likely ‘blunted’ differences observed between the cohorts [6]. Further studies have explored the pivotal trials and complete dossiers of orphan medicinal products approved by the European Medicines Agency (EMA); although neither publications provided contrasting characteristics for non-orphan products [7,8]. To our knowledge no large scale comparative survey has been undertaken to contrast rare disease trials with those in other conditions.

ClinicalTrials.gov provides a public access registry and results database recording clinical studies on human participants. It provides a global registry of both publicly and privately supported trials. The registry is widely used currently containing close to 150,000 research studies. Data can be downloaded across all registered studies and has been used to explore various characteristics of clinical trials [9,10]. Recently, the usability of the ClinicalTrials.gov dataset has been extended for research purposes through the development of the Aggregate Analysis of ClinicalTrials.gov (AACT) database [11]. Research using the AACT database to conduct surveys of clinical trials across different clinical specialties is now emerging. Particular examples include Califf *et al*. who provide specific characterisations of interventional trials in the areas of oncology, cardiovascular and mental health and Hirsch *et al*. who also characterise interventional oncology trials with further sub-categorisation [12,13].

Our objectives were to use the AACT database to provide a comprehensive characterisation of rare disease clinical trials registered in ClinicalTrials.gov. Further, we wished to compare these characteristics against those of trials in other, non-rare, conditions.

## Methods

### The AACT database

On July 25, 2013 we downloaded the 2012 AACT database from the CTTI website [14]. The database comprised of 133,128 clinical studies registered with ClinicalTrials.gov to September 27, 2012. The key feature of the design, structure and purpose of the AACT database are detailed in Tasneem *et al.* and the corresponding website [11,14].

When submitting trials to ClinicalTrials.gov data submitters are requested to provide the diseases and clinical conditions under study as Medical Subject Heading (MeSH) terms. Further, ClinicalTrials.gov uses an algorithm provided by the National library of Medicine (NLM) to annotate additional MeSH terms to a given study [11].

The same condition may exist in ClinicalTrials.gov under various synonyms. To help categorise conditions we downloaded the 2013 MeSH thesaurus and the 2013 supplementary concept records [15,16]. Each record within these sources contained a MeSH Id, a preferred descriptor name and the names of related concepts which provided synonyms and lexical variations of the preferred descriptor. Table 1 shows an example for Ewing Sarcoma of the information extracted from these sources. In this case ‘Sarcoma, Ewing’ is the preferred MeSH descriptor and the remaining entries are potential variations all mapped to the same MeSH Id.

**Table 1:** **Example of the MeSH database table used to identify conditions**

**ROW_ID**	**MESH_TERM**	**MESH_ID**
1	Sarcoma, Ewing	D012512
2	Ewing Sarcoma	D012512
3	Ewing's Tumor	D012512
4	Sarcoma, Ewing's	D012512

For the 133,128 clinical studies in the AACT database a total of 211,063 user submitted conditions had been provided. Of these 144,871 (68.6%) could be annotated with a MeSH Id. A further 268,796 MeSH terms had been annotated to these trials by the NLM algorithm; these were also matched to their relevant MeSH Id.

### Identification of rare diseases

To subgroup studies into rare and non-rare diseases it remained to identify MeSH Id’s related to rare diseases. To identify these we consolidated information from three sources. Firstly, ClinicalTrials.gov identifies trials involving rare diseases within the ‘By Topic’ display option. This listed a total of 1,430 rare diseases based on the US definition. Secondly, we downloaded documentation entitled ‘Rare Disease and Cross References’ from OrphanData.org containing 6,767 diseases classified as rare within the EU [17]. As a final resource we extracted rare conditions from the alphabetical lists provided by the Office of Rare Diseases Research [18]. This provided an additional 6,526 rare disease condition names. Upon removing duplicates a total of 11,959 rare disease names, synonyms and lexical variations remained. Using a combination of lookups, pattern matching and manual search a total of 6,389 (54%) rare disease names, synonyms and lexical variations were matched to their relevant MeSH Id. In total 4,516 unique MeSH Id’s were used and identified as rare. Whilst 54% may seem a low percentage, many of the 11,959 diseases listed are extremely rare and only recently identified. For quality assessment purposes we performed a Targeted Search of ClinicalTrials.gov using each of the 11,959 disease names as conditions. The total number of studies returned from each search was recorded. For example a targeted search using ‘Krabbe disease’ as the condition name returned 19 studies that may relate to this disease. In total 194,226 studies were returned via this search method, of which, 177,333 (91%) where contained within the 6,389 of conditions we identified as having MeSH Id’s.

### Creation of data sets for comparison

**Figure 1:**
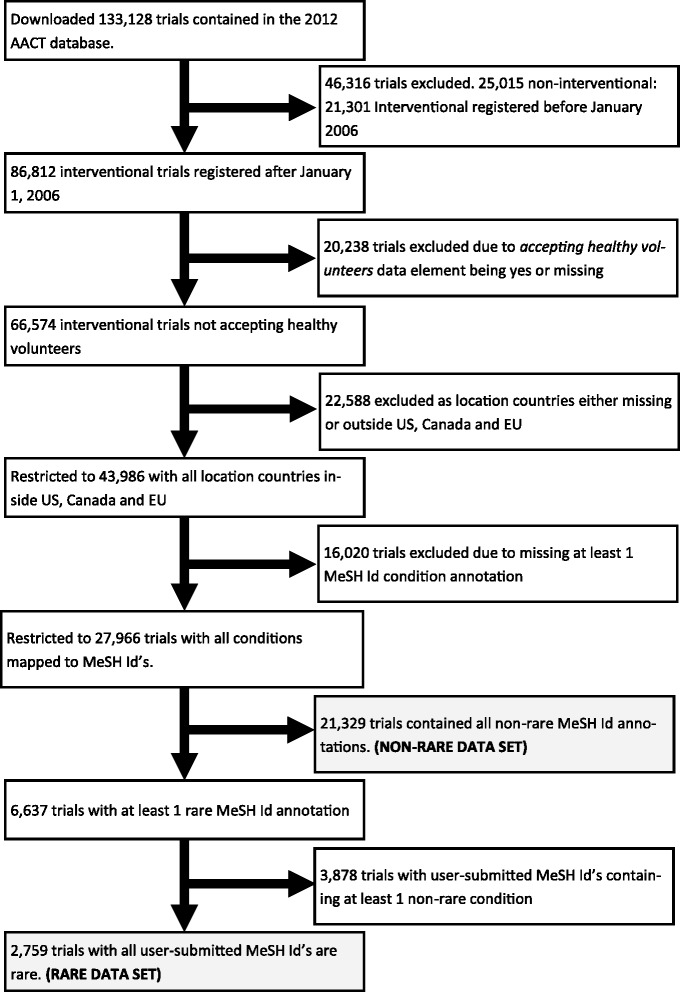
**Identification of the comparison data sets (shaded boxes show the final data sets used in analysis.**

Final datasets for comparison (Figure 1) were created using the annotated MeSH Id’s following a number of restrictions. Briefly, of the 133,128 studies in the database only interventional studies were eligible for inclusion leaving 108,113 studies. Attention was restricted to trials first registered on or after January 1, 2006 leaving 86,812 studies.

Our goal was to characterise trials on patients with rare diseases thus any trials accepting healthy volunteers were excluded leaving 66,574 studies. Finally, the conditions identified as rare are only rare in specific countries. We excluded any trial with participating centres registered outside the US, EU or Canada leaving 43,986 studies, of these 27,966 trials had a complete complement of MeSH Id’s allowing for categorisation.

A trial was identified as a non-rare disease trial if none of its annotated MeSH Id’s had been identified as rare. A total of 21,329 trials had no rare MeSH Id’s; 6,637 trials had at least 1 rare disease MeSH Id. Trials were finally classified as rare based on the user submitted MeSH Id’s only. We classified a trial as a rare disease trial if all user submitted MeSH Id’s were rare; this left 2,759 rare disease trials.

### Analytical methods

Prior to analysis certain missing characteristics were inferred. When a trial relates to a single group with the interventional model described as *single assignment* the trial was designated as non-randomised with open blinding [12].

Descriptive statistics were used to characterise the identified datasets. For categorical data, frequencies and percentages are stated; for continuous data, medians and interquartile ranges (IQR) are stated. We did not have any pre-specified hypotheses in this study and have not presented results from statistical tests. Between group differences in percentages are presented for categorical characteristics. The 95% confidence intervals for the between group difference was calculated using the Wilson procedure without continuity correction. R 2.13 was used for all statistical analysis [19].

### Ethical approval

This analysis of existing publicly available data relates to clinical trials and their characteristics rather than human participants. Ethical approval was not required.

## Results

From January 1, 2006 to September 27, 2012 a total of 2,759 trials were identified as relating to rare diseases and 21,329 trials as relating to non-rare diseases. The 2,759 rare disease trials include only 430 unique MeSH Id’s for the conditions under study. This corresponds to only 9.5% (430/4516) of the rare disease unique MeSH Id’s identified. The labels used in the clinicaltrials.gov database are in some sense weakly defined and can often be interpreted differently by different users which might impact the validity of results presented here.

### Trial characteristics

Basic characteristic of trials in the identified datasets are shown in Table 2. The meaning of characteristics is generally self-evident, although exact definitions for certain characteristics are given in the text. Further definitions of characteristics can be found in the ClinicalTrials.gov draft Protocol Data Element Definitions and e-appendix 1 of Califf *et al.* [12,20].

**Table 2:** **Characteristic of rare and non-rare disease clinical trials within the ClinicalTrials.gov registry**

**Characteristics**		Rare disease trials* (n = 2759)	Non-rare disease trials* (n =21329)	**Difference in percentage points (rare–non-rare)**	**95% CI for difference**
**Year enrolment to protocol began, n (%)**					
	Prior to 2006	455 (16.7)	2791 (13.2)	3.4	2 to 5
	2006-2007	871 (31.9)	5121 (24.2)	7.6	6 to 10
	2008-2009	859 (31.5)	5858 (27.8)	3.7	2 to 6
	2010-2011	467 (17.1)	5315 (25.2)	−8.1	−7 to −10
	2012 and after	78 (2.9)	2018 (9.6)	−6.7	−6 to −7
**Overall status, n (%)**					
	Completed	1169 (42.4)	8564 (40.2)	2.2	0 to 4
	Not yet recruiting	15 (0.5)	1030 (4.8)	−4.3	−4 to −5
	Recruiting	372 (13.5)	6840 (32.1)	−18.6	−17 to −20
	Withdrawn	99 (3.6)	332 (1.6)	2.0	1 to 3
	Active, not recruiting	638 (23.1)	2742 (12.9)	10.3	9 to 12
	Terminated	379 (13.7)	1332 (6.2)	7.5	6 to 9
	Suspended	36 (1.3)	154 (0.7)	0.6	0 to 1
	Enrolling by invitation	51 (1.8)	335 (1.6)	0.3	0 to 1
**Gender, n (%)**					
	Female	116 (4.2)	1949 (9.1)	−4.9	−4 to −6
	Male	60 (2.2)	1042 (4.9)	−2.7	−2 to −3
	Both	2583 (93.6)	18338 (86.0)	7.6	7 to 9
	**Includes paediatric (<18), n (%)**				
	Yes	546 (20.6)	2294 (11.1)	9.5	8 to 11
	No	2106 (79.4)	18320 (88.9)	−9.5	−8 to −11
	**Includes Elderly (>65), n (%)**				
	Yes	2291 (86.4)	17157 (83.2)	3.2	2 to 5
	No	361 (13.6)	3457 (16.8)	−3.2	−2 to −5
**Anticipated enrolment‡, n (%)**					
	0-50	798 (61.7)	4556 (38.2)	23.5	21 to 26
	51-100	280 (21.6)	2731 (22.9)	−1.3	0 to −4
	101-500	195 (15.1)	3767 (31.6)	−16.5	−14 to −19
	500+	21 (1.6)	877 (7.4)	−5.7	−5 to −6
**Actual enrolment‡, n (%)**					
	0-50	955 (71.4)	3570 (43.3)	28.1	25 to 31
	51-100	211 (15.8)	1607 (19.5)	−3.7	−1 to −6
	101-500	158 (11.8)	2402 (29.1)	−17.3	−15 to −19
	500+	14 (1.0)	672 (8.1)	−7.1	−6 to −8
**Lead Sponsor, n (%)**					
	Industry	951 (34.5)	6437 (30.2)	4.3	2 to 6
	NIH	72 (2.6)	602 (2.8)	−0.2	0.5 to −0.8
	US Federal	15 (0.5)	472 (2.2)	−1.7	−1 to −2
	Other	1721 (62.4)	13817 (64.8)	−2.4	−1 to −4
**Location, n (%)**					
	US only	1720 (62.3)	12073 (56.6)	5.7	4 to 8
	Canada only	83 (3.0)	1387 (6.5)	−3.5	−3 to −4
	EU only	733 (26.6)	6938 (32.5)	−6.0	−4 to −8
	US/Canada	84 (3.0)	405 (1.9)	1.1	1 to 2
	US/EU	93 (3.4)	333 (1.6)	1.8	1 to 3
	EU/Canada	9 (0.3)	55 (0.3)	0	−0.1 to 0.4
	US/EU/Canada	37 (1.3)	138 (0.6)	0.7	0.3 to 1
	**Number of countries, n (%)**				
	Single country	2442 (88.5)	19847 (93.1)	−4.5	−3 to −6
	2-3 countries	219 (7.9)	1057 (5.0)	3.0	2 to 5
	≥4 countries	98 (3.6)	425 (2.0)	1.6	1 to 2

*Denominators exclude missing values. Missing data values [Rare (%), Non-Rare(%)] are: Year of enrolment [29(1.1),226(1.1)]; Study duration [361(13.1),2784(13.1)]; Ages [107(3.9),715(3.3)]; Enrolment [127(4.6),1147(5.4)].

‡Anticipated and Actual enrolment are mutually exclusive in ClinicalTrials.gov (Rare Anticipated [1294] vs. Actual [1338]; Non-Rare Anticipated [11931] vs. Actual [8251]).

The proportion of trials which are in rare diseases has decreased over time based on the year enrolment to protocol began (Table 2). The overall recruitment status also suggests a higher proportion of rare disease trials are active but not yet recruiting or have terminated early with fewer currently recruiting.

Study duration is defined as the date from which enrolment begins until the final date on which data was, or is anticipated to be, collected. The median study duration, in years, is longer in rare disease trials (median, 3.2[IQR 2.0-4.9] vs. 2.3[1.3, 3.8]).

Participant eligibility characteristics based on gender and age are more inclusive in rare disease trials with a higher proportion of rare disease trials that included both genders (93.6% vs. 86.0%), paediatric patients (20.6% vs. 11.1%) and elderly patients (86.4% vs. 83.2%). It should be noted that this is only enrolment criteria and not data on those who actually participated.

In ClinicalTrials.gov a trial can report either actual or expected numbers recruited but not both. The anticipated number of patients to be recruited in rare trials is less than that of non-rare disease trials (median, 41[IQR 24–74] vs. 76[40–182]). Similarly, the actual number of patients recruited is less for rare disease trials than in non-rare trials (29[12–60] vs. 62[27–175]). For rare disease trials the median actual enrolment is 70.1% (29/41) of the median anticipated enrolment compared to 81.6% (62/76) for non-rare trials and the proportion of trials in rare diseases decreases as enrolment number (anticipated or actual) increases.

The proportion of rare disease trials with an industrial lead sponsor, defined as ‘the organization or person who oversees the clinical study and is responsible for analysing the study data’. ClinicalTrials.gov [20], is slightly greater (34.5% vs. 30.2%) than non-rare disease trials. The majority of trials of either type are undertaken within a single administrative region (US, Canada or the EU). However, a higher proportion of rare disease trials are undertaken across these administrative regions (8.1% vs. 4.4%) compared to non-rare disease trials. Further, exploring individual countries as opposed to these administrative regions, a higher proportion of rare disease trials reported being multi-national (11.5% vs. 6.9%).

### Design characteristics

Design characteristics of rare diseases trials differ to non-rare diseases trials (Table 3). Centres (labelled as facilities in ClinicalTrials.gov) identify where the protocol is being conducted. A lower proportion of rare disease trials conducted the study protocol at a single centre compared to non-rare trials (61.3% vs. 72.7%).

**Table 3:** **Design attributes of clinical trial within the ClinicalTrials.gov data set**

**Characteristics**		Rare disease trials* (n = 2759)	Non-rare disease trials* (n = 21329)	**Difference in percentage points (rare –non-rare)**	**95% CI for difference**
**Number of facilities, n (%)**					
	Single facility	1671 (61.3)	15443 (72.7)	−11.4	−9 to −13
	2-3 facilities	333 (12.2)	2482 (11.7)	0.5	0 to 2
	≥4 facilities	720 (26.4)	3315 (15.6)	10.8	9 to 13
**Study phase, n (%)**					
	Phase 0	15 (0.5)	166 (0.8)	−0.2	0 to −0.5
	Phase 1	410 (14.8)	1848 (8.7)	6.2	5 to 8
	Phase 1/2	294 (10.7)	1014 (4.8)	5.9	5 to 7
	Phase 2	1212 (43.9)	4747 (22.3)	21.7	20 to 24
	Phase 2/3	83 (3.0)	601 (2.8)	0.2	0 to 1
	Phase 3	287 (10.4)	3110 (14.6)	−4.2	0 to −5
	Phase 4	145 (5.3)	3398 (15.9)	−10.7	−10 to −12
	NA	313 (11.3)	6445 (30.2)	−18.9	−17 to −20
**Primary purpose, n (%)**					
	Treatment	2451 (91.2)	16195 (79.3)	12.0	11 to 13
	Prevention	87 (3.2)	1488 (7.3)	−4.0	−3 to −5
	Screening	1 (0.0)	86 (0.4)	−0.4	−0.2 to −0.5
	Supportive Care	39 (1.5)	844 (4.1)	−2.7	−2 to −3
	Health Services Research	7 (0.3)	446 (2.2)	−2	−2 to −2
	Educational/Counselling/Training	2 (0.1)	14 (0.1)	0.0	0 to 0
	Diagnostic	75 (2.8)	917 (4.5)	−1.7	−1 to −2
	Basic Science	25 (0.9)	441 (2.2)	−1.2	−1 to −2
**Study has DMC†, n (%)**					
	Yes	1211 (53.2)	7274 (40.9)	12.3	10 to 14
	No	1066 (46.8)	10511 (59.1)	−12.3	−10 to −14
**Intervention model¥, n (%)**					
	Single Group Assignment	1691 (63.0)	6134 (29.6)	33.5	32 to 35
	Parallel Assignment	837 (31.2)	12466 (60.1)	−28.9	−27 to −31
	Crossover Assignment	140 (5.2)	1741 (8.4)	−3.2	0 to −4
	Factorial Assignment	14 (0.5)	411 (2.0)	−1.5	−1 to −2
**Intervention type‡, n (%)**					
	Drug	2204 (79.9)	11891 (55.8)	24.1	22 to 26
	Device	127 (4.6)	2620 (12.3)	−7.7	−7 to −9
	Procedure	269 (9.7)	2408 (11.3)	−1.5	0 to −3
	Biological	220 (8.0)	926 (4.3)	3.6	3 to 5
	Radiation	112 (4.1)	476 (2.2)	1.8	1 to 3
	Behavioural	47 (1.7)	2631 (12.3)	−10.6	−10 to −11
	Dietary Supplement	31 (1.1)	562 (2.6)	−1.5	−1 to −2
	Genetic	39 (1.4)	124 (0.6)	0.8	0 to 1
	Other	176 (6.4)	2700 (12.7)	−6.3	−5 to −7
**Allocation¥, n (%)**					
	Randomised	949 (35.5)	14958 (71.6)	−36.1	−34 to −38
	Non-randomised	1727 (64.5)	5937 (28.4)	36.1	34 to 38
**Endpoint classification, n (%)**					
	Efficacy Study	530 (22.0)	7454 (42.0)	−20.0	−18 to −22
	Safety/Efficacy Study	1521 (63.2)	8148 (45.9)	17.3	15 to 19
	Safety Study	276 (11.5)	1154 (6.5)	5.0	4 to 6
	Pharmacodynamics Study	9 (0.4)	282 (1.6)	−1.2	−0.8 to −1.5
	Pharmacokinetics Study	36 (1.5)	339 (1.9)	−0.4	0.2 to −0.9
	Pharmacokinetics/Dynamics Study	27 (1.1)	234 (1.3)	−0.2	0.3 to −0.5
	Bio-equivalence Study	5 (0.2)	90 (0.5)	−0.3	0 to −0.5
	Bio-availability Study	1 (0.0)	47 (0.3)	−0.2	−0.2 to −0.3
**Type of arms‡, n (%)**					
	Experimental	1743 (84.3)	13063 (73.4)	10.4	9 to 12
	Active comparator	458 (22.2)	7569 (42.8)	−20.7	−19 to −23
	Placebo comparator	343 (16.6)	4701 (26.6)	−10.0	−8 to −12
	Sham comparator	6 (0.3)	346 (2.0)	−1.7	−1 to −2
	No Intervention	73 (3.5)	1908 (10.8)	−7.3	−6 to −8
	Other	84 (4.1)	111 (6.3)	3.4	3 to 4
**Blinding, n (%)**					
	Open	2137 (78.7)	10967 (52.2)	26.6	25 to 28
	Single	89 (3.3)	2680 (12.8)	−9.5	−9 to −10
	Double	488 (18.0)	7370 (35.1)	−17.1	−15 to −19

*Denominators exclude missing values. Missing data elements [Rare(%), Non-Rare(%)]: No. of Facilities [35(1.3),89(0.4)]; Primary purpose [72(2.6),898(4.2)]; Study has DMC [482(17.5),3544(16.6)]; Intervention model [77(2.8),577(2.7)]; Allocation [83(3.0),434(2.0)]; Endpoint Classification [354(12.8),3581(16.8)]; Type of arms [692(25.1),3650(17.1)]; Blinding [45(1.6),312(1.5)].

†Data Monitoring Committee.

‡Studies can belong to multiple categories.

¥42% of studies labelled as single arm trials were missing allocation information. Whilst some missing data is inferred (see methods section) the trials still missing allocation information are predominantly single-arm trials but missing group data. This allows there to be more single group assignment trials than there are non-randomised trials for the non-rare disease dataset.

Rare disease trials are more likely to be early phase trials (those reporting a phase 1 and/or 2 element), (72.5% vs. 38.5%). A lower proportion of rare disease trials employed the N/A phase description (11.3% vs. 30.2%) which is advised to be used for trials that do not involve drug or biologic products although this is not enforced in ClinicalTrials.gov [20]. The primary purpose of a rare disease trial is generally treatment (91.2% vs. 79.3%) with the intervention used tending to be a drug (79.9% vs. 55.8%).

The appointment of a Data monitoring committee (DMC) is more common in rare disease trials (53.2% vs. 40.9%). They are also more likely to have a single group assignment (63.0% vs. 29.6%), with no randomisation (64.5% vs. 28.4%) and open label (78.7% vs. 52.2%). Rare disease trials are also more likely to explore both safety and efficacy endpoints in the same trial (63.2% vs. 45.9%).

## Discussion

This analysis of ClinicalTrials.gov suggests that trials in rare diseases differ from those of non-rare conditions. Rare disease trials are more likely to have smaller target sample size, more likely to be early-phase, more likely to recruit to a single arm, more likely to be non-randomised, and more likely to be unblinded. These results support those of Kesselheim *et al.* and Mitsumoto *et al.* that have explored orphan drug approval trials in the specialities of cancer and neurology respectively [4,21]. Results from our study add to current knowledge by including a wider array of clinical studies, not just those completed and used in application for drug approval, and by examining a number of characteristics that have not been considered previously.

It has been argued that whenever possible standard methodological approaches, such as the randomised controlled trial, should be applied in the design and analysis of a clinical trial [22]. However, due to the very limited pool of eligible patients with rare diseases, evidence from good quality randomised controlled trials is often absent for a particular disease. The rarity of the disease makes enrolling a sufficient number of participants inherently difficult.

A higher proportion of rare disease trials employ a multi-centre design; potentially across multiple countries. However, whilst there is an increased use of multi-centre trials in rare diseases compared to non-rare diseases, they are still predominantly single centred. This suggests there is potential for important improvements to be made in the numbers of patients recruited to rare disease trials. Further cooperation between research facilities could greatly improve research and the on-going development of patient contact registries could also serve as powerful tools for improving recruitment [23].

A larger proportion of rare disease trials enrolled paediatric participants particularly in conjunction with adult participants. That 67.9% of rare disease trials within the orpha.net database had an age of onset given as neonatal, infancy or childhood could go some way to explaining this result. In general, eligibility criteria in rare disease trials were less restrictive than in non-rare trials. Whilst we only have eligibility criteria in terms of age and gender in the ClinicalTrials.gov dataset the idea of the introduction of participant heterogeneity needs to be carefully considered in the analysis plan of any rare disease trials. Failure to identify the most appropriate target population was found to be a key feature of failed orphan marketing authorisations in both the EU and US [24,25].

Despite these attempts to improve participation rare disease clinical trials are still frequently small, single arm studies. Concerns have been raised over the quality of evidence obtained from such studies [26,27]. It is argued that even in rare disease trials, where we cannot provide a large quantity of evidence, researchers should still attempt to provide good quality evidence [26]. Blinded, randomised controlled trials are classed as the gold standard for clinical trials. Whilst not straight-forward to perform in rare diseases, these standards can still often be achieved. Recent reviews have summarised many alternative trial designs that can provide adequate and well-controlled data for rare disease trials [28,29]. Of course, design recommendation for rare disease trials such as Bayesian methods [30] and adaptive randomisation [31] are subtle and not identifiable in ClincalTrials.gov and further analysis of published studies would be required to explore if such recommendations have been implemented.

A higher proportion of rare disease trials explored drug and biological interventions than non-rare diseases trials. One possible explanation is the 1983 Orphan Drug Act (ODA) in which the term "orphan drug" refers to a drug or biologic. It has been highlighted that the ODA provides a shelter for many biologic treatments as market exclusivity provides a surrogate form of patent. Other legislation, such as the Humanitarian Device Exemption within the Safe Medical Devices Act of 1990, exists to support development of other interventions in rare diseases [32]. Despite these legislative incentives few researchers have explored alternative interventions through clinical trials.

The overall status of rare disease trials shows a proportionately larger number have been terminated, withdrawn or suspended. A comprehensive analysis of why this is the case would be a useful aid for future researchers planning rare disease clinical trials and potentially prevent wasted resources. It is estimated that only 10% of rare diseases have an available treatment [33]. In fact, in our study we found that only 9.5% of possible ‘rare disease’ MeSH terms identified had an associated clinical trial which corresponds well with this finding. Even for rare diseases with licenced orphan medicinal products, these treatments can still be improved and there have been concerns expressed regarding the clinical evidence and lack of efficacy data for these products [8,34]. Joppi *et al.* reflect that “…nearly all the currently estimated 7,000 rare diseases, with approximately 250 new diseases described annually, still await treatment”, as such, the medical need for appropriate clinical research in rare diseases remains high.

### Limitations

Numerous limitations to this study need to be noted. Firstly our categorisation of rare and non-rare trials relied on conditions being submitted as MeSH terms. Whilst this is recommended by ClinicalTrials.gov, it is not enforced. Approximately 30% of studies were instantly excluded because we could not map conditions to MeSH Id’s. Of the 11,959 rare disease terms identified, we could only match 54% to MeSH Id’s. Better application of text matching routines and the use of clinical experts could improve this percentage. A potential consequence is that a number of false negative results may be present in the non-rare disease trial dataset; their correct categorisation could help improve the robustness of this characterisation study. However, taking a random sample of 50 trials from the non-rare dataset we did not find any false-negative results. Thus, we believe that this is unlikely to introduce any bias into the finding. The use of standard vocabulary for conditions submitted to ClinicalTrials.gov could facilitate future research.

Using both US and EU rare disease sources may also limit this study as definitions of rare disease differ in these regions. However, there is likely a large crossover of conditions regardless of regions. Taking a random sample of 50 trials from the rare disease data set found only 4 (8%) trials that were not listed as rare in both the US and EU. These 4 trials listed the conditions of epithelial ovarian cancer, compartment syndrome, photosensitive epilepsy and focal segmental glomerulosclerosis. They are all listed as rare in the ClinicalTrials.gov registry but prevalence for these conditions was not confirmed in the EU orpha.net database [3].

Califf *et al*. describe a number of limitations to the ClinicalTrials.gov dataset [12]. Firstly, ClinicalTrials.gov does not register all trials. Registries such as ClinicalTrialsRegister.eu can also be used. Confirmation of temporal trends in the ClinicalTrials.gov dataset may need to be corroborated across registries. This is particularly true for specialist datasets; if large research institute register future studies in a different database this could have large implications on the identification of trends.

Secondly, data elements can be missing or unavailable due to changes in the ClinicalTrials.gov registry definitions, statutory requirements or simply not entered by data submitters. Further to missing data elements, data provided can also be inconsistent or inaccurate. For example a number of trials reported with single arm assignment had also reported a crossover, factorial or parallel intervention model. Furthermore, we did not explore the details behind the labels provided for each trial and we recognise that there may be inaccuracies in definitions such as ‘randomized’ or ‘blinded’. Causes for this may be the expertise of data submitters or a lack of suitable constraints on the data being submitted to ClinicalTrials.gov. Missing data, inconsistencies, and inaccuracies can impact the quality of this, and any other, characterisations of trials. However, we believe that these issues are likely to introduce random error rather than bias since the issues are likely to be distributed similarly across rare and non-rare disease trials.

It is also worth pointing out the strengths of this analysis in that we have provided one of the largest and most complete comparative surveys of rare disease clinical trials to date.

## Conclusion

ClincalTrials.gov and the AACT database can help us characterise clinical research in rare diseases. With the Food and Drug Administration (FDA) and EMA providing significant support for rare disease research it is critical to understand how research is implemented to help identify where potential improvements can be made. The ClinicalTrials.gov dataset shows that trials in rare diseases use fewer participants and have longer duration as might be expected. However, our study also shows that rare disease trials are less likely to use blinding and randomisation than trials in other areas. These are generally regarded as hallmarks of high-quality clinical trial design and this raises concerns over the quality of evidence being supplied by rare disease trials. With higher termination rates for these trials, additional research is required to identify ways to improve the quality, as well as the quantity of rare disease trials.

### What this paper adds

#### What is already known on this topic?

The evaluation of treatments for rare diseases presents a number of challenges for trial practitioners, regulators and policy makers. Within certain clinical specialities rare disease trials have been found to be smaller and to tend to use nonrandomized, unblinded designs compared to trials in non-rare conditions.

### What this study adds

This study contrasts rare and non-rare diseases clinical trials across a broader range of characteristics than previously seen in the literature. Trials in rare and non-rare conditions differ in methodological approach including quantifiable differences in the reported use of randomisation, blinding and data monitoring committees. Rare disease trials are smaller, longer and more frequently terminated than those in non-rare conditions.

### Availability of supporting data

Data used for this study are available from the following website http://www.ctti-clinicaltrials.org/what-we-do/analysis-dissemination/state-clinical-trials/aact-database or can be obtained by contacting the corresponding author.

## Abbreviations

AACT: Aggregate Analysis of ClinicalTrials.gov
DMC: Data monitoring committee
FDA: Food and Drug Administration
EMA: European Medicines Agency
EU: European Union
IQR: Interquartile Range
MeSH: Medical Subject Heading
NLM: National library of Medicine
ODA: Orphan Drug Act
US: United States
